# Firmware Update Using Multiple Gateways in LoRaWAN Networks

**DOI:** 10.3390/s21196488

**Published:** 2021-09-28

**Authors:** Christia Charilaou, Spyros Lavdas, Ala Khalifeh, Vasos Vassiliou, Zinon Zinonos

**Affiliations:** 1 Department of Computer Science, University of Cyprus, Nicosia 1678, Cyprus; cchari03@cs.ucy.ac.cy (C.C.); vasosv@cs.ucy.ac.cy (V.V.); 2 Department of Computer Science, Intelligent Systems Laboratory, Neapolis University Pafos, Pafos 8042, Cyprus; s.lavdas@nup.ac.cy; 3 Electrical Engineering Department, German Jordanian University, Amman 11180, Jordan; ala.khalifeh@gju.edu.jo; 4 CYENS Center of Excellence, Nicosia 1016, Cyprus

**Keywords:** firmware update, LoRaWAN, multiple gateways, simulation

## Abstract

The remarkable evolution of the IoT raised the need for an efficient way to update the device’s firmware. Recently, a new process was released summarizing the steps for firmware updates over the air (FUOTA) on top of the LoRaWAN protocol. The FUOTA process needs to be completed quickly to reduce the systems’ interruption and, at the same time, to update the maximum number of devices with the lowest power consumption. However, as the literature showed, a single gateway cannot optimize the FUOTA procedure and offer the above mentioned goals since various trade-offs arise. In this paper, we conducted extensive experiments via simulation to investigate the impact of multiple gateways during the firmware update process. To achieve that, we extended the FUOTAsim simulation tool to support multiple gateways. The results revealed that several gateways could eliminate the trade-offs that appeared using a single gateway.

## 1. Introduction

Internet of Things (IoT) has already been incorporated in various fields such as manufacturing, health care, security, and more, offering intelligent services to several users. Consequently, new smart environments were created, such as smart homes and smart cities, where many IoT applications [1,2] were created to support this evolution. The growth of IoT is estimated to increase dramatically into the near future, aiming to create a new digital world.

In order to support this progression and accomplish useful IoT deployments, the devices’ firmware must be upgraded to fix bugs and add more security features, optimizations, and functionalities. In that case, devices would be more secure, their lifetime would be expanded, and systems’ performance would be improved. However, firmware update is considered a challenge in IoT since oftentimes there is a large number of devices and/or devices located in remote or hard-to-reach areas where their software must be updated manually. In consequence, this human intervention is strenuous enough to cause network degradation, which often leads to network system interruption. Nevertheless, the IoT’s remarkable evolution increases the need to create new remote firmware update techniques to avoid flustering or degrading the system’s performance. Recently, a new solution has been proposed, the Firmware Update over the Air (FUOTA), which can update the IoT devices’ firmware remotely using the wireless medium [3].

Supporting the functionality of FUOTA in the IoT communication protocol LoRaWAN can extend the promised devices’ lifetime, enhance their performance, and achieve an efficient IoT deployment [4]. Based on FUOTA requirements, the gateway needs to send hundreds of bits to the end devices as the firmware image consists of several kilobytes. Given that the LoRaWAN protocol was primarily designed for low data rate applications that send up-link data infrequently in a wide area, it is considered a significant challenge to support the FUOTA procedure. Hence, to complete the update successfully the firmware image must break into equal fragment sizes that support the payload limitation. To give an illustration, transmitting a 100 kbytes firmware image to the end devices using DR0 mode (SF12, BW125 kHz) would require 2008 downlink packets since the payload size is limited to 51 bytes. Furthermore, the LoRaWAN data rate limitations (usually 1% duty cycle in the EU) make the firmware update procedure even more challenging as devices transmit data only in a fraction of time. Since the protocol operates in the license-free sub-1 GHz ISM bands, it can experience interference and thus packet losses. However, the firmware image must be received unchanged and, therefore, a new mechanism to recover from packet losses without re-transmitting them is essential. Since these limitations were preventing the efficient use of FUOTA in LoRaWAN, the Lora Alliance has proposed new specifications to support multicasting, fragmentation, and clock synchronization, which are essential features to enable FUOTA in the LoRaWAN [4].

The FUOTA process can be evaluated in terms of firmware update time and update efficiency, which indicates the number of devices that updated their firmware successfully. Another metric that is critical for the firmware update process is energy consumption. Therefore, to mitigate the system’s disruption and attain maximum performance, the FUOTA process must be completed with maximum update efficiency, shortest update time, and lowest energy consumption.

In many scenarios, especially in the Smart City context, the number of end-nodes devices is expected to be high and the number of gateways is of utmost importance to achieve the required performance during the firmware update procedure. It is confirmed from the literature [5,6], that using GW diversity in a large area can improve the overall coverage, reduce the overhead, and increase the reliability and scalability. Hence, considering the aforementioned benefits, it is expected that GW diversity will have an enormous impact on the FUOTA procedure as well. To the best of our knowledge, this is the first scientific paper that considers the impact of multiple GWs during the firmware update process as currently in the literature, only evaluations using a single gateway are presented.

Our contributions in this work are three-fold:

1. We conduct extensive experiments via simulation to investigate the impact of multiple gateways along with varying network and firmware sizes to observe and analyze the network’s behavior during the firmware update process.
2. We investigate the minimum set of gateways that can be used to provide full coverage with the greatest performance during the firmware update procedure, while at the same time maintaining acceptable operational and deployment costs.
3. We offer useful insights into the tradeoffs between the number of gateways, the firmware size, and the communication characteristics (spreading factors and forward error correction mode).

The remainder of the paper is organized as follows. Section 2 briefly presents the related work about firmware updates and different gateway placement techniques. The FUOTA process using multiple gateways is discussed in Section 3 along with the firmware update scenario, propagation model, and creation of multicast groups. Section 4 contains an evaluation of the proposed solution and a presentation of the collected results. In Section 5 authors present some further conclusions from the simulation results and discuss future work. Finally, the conclusions of the study are summarized in Section 6.

## 2. Related Work

The IoT deployment evolution raised the need for an effective way to update the IoT devices’ embedded software. Therefore, the innovative solution for firmware updates over the air (FUOTA) attracted many researchers’ attention. Current investigations cover many aspects of the FUOTA process, such as the security of performing the process, its impact on LoRaWAN protocol, the importance of remote firmware updates, and different trade-offs between update efficiency, data rate, and energy consumption.

In [7] the challenges and solutions for implementing FUOTA in LPWAN networks are presented. The need to reliably update multiple devices simultaneously, considering any packet losses, is the primary key to effectively accomplishing FUOTA.

In [8] authors describe the significance of updating the IoT devices’ firmware. IoT devices are susceptible to attacks when their firmware contains vulnerabilities and cannot be updated automatically. Consequently, this can lead to losing control of the system and also to system relegation. Further, they highlight security problems and challenges that IoT-constrained devices and LPWAN will face during the remote firmware update.

Therefore, preserving the integrity and security of the firmware image is another challenge in IoT and FUOTA. Many researchers used the blockchain solution to ensure security in the FUOTA process. In [9] authors created a blockchain framework with smart contracts to accomplish it. A system was implemented and assessed using this approach. Their evaluation has shown that the system operates well and can be used as a solution. To continue with, in [10] authors proposed a mechanism using the blockchain to improve the firmware update process in LoRaWAN.

Authors in [11] also used the LoRaWAN protocol along with a blockchain solution to increase the security when the firmware is updating through the air. They also proposed a blockchain-based framework where their evaluation has shown that using only one gateway node leads to different trade-offs between firmware update size, data rate, and network scalability. Hence, they concluded that there is a need to manage the above-mentioned trade-offs and improve the reliability and system’s performance during the FUOTA process. The authors came to the point that the collaboration of several GWs is a practical way to accomplish that.

In [4] the FUOTA process is described and analyzed through a simulation tool. They separated the FUOTA process into two phases to observe and note its behavior. Furthermore, they assessed the impact of different FUOTA parameters such as the update efficiency, update time, and network energy using different LoRaWAN parameters. They used two LoRaWAN protocol classes (B and C), all achievable data rates, and a single GW and studied the results. Their evaluation showed the significant impact of the data rates and the nodes’ distribution on the FUOTA process. Higher data rates lead to considerably lower update time and energy consumption. However, it comes at the cost of the update efficiency [4], where only a small subsection of the devices successfully update their firmware if the distance exceeds the transmission range. On the contrary, lower data rates update a higher subgroup of devices due to the greater noise immunity but result in much higher update time and energy consumption. Nevertheless, the authors concluded that the GW spatial diversity could eliminate the previous trade-offs if the GWs are distributed to reach all nodes with the highest data in the license-free sub-1GHz ISM bandrate.

Therefore, as literature showed, many authors concluded that multiple GWs are a novel solution to achieve best performance when using the FUOTA in LoRaWAN since they seem to abate the previous trade-offs and enhance the operation of the system. However, none of them provide further insights on the interplay of the GW diversity in the FUOTA process. This work aims to fill the particular lack of a comprehensive investigation of the matter. To achieve that we extended the FUOTAsim simulation tool to support the multiple GWs scenario during the firmware update.

To include multiple gateways in a LoRaWan network is considered as another provoking task. Gateways must provide full coverage, consider any possible limitations due to duty cycle or collisions, and be able to provide best performance by allowing the use of the lowest SF. It is also important to use the minimum set of gateways in order to reduce utmost the cost deployment. Hence, many researchers considered the aforementioned problems and came up with different placement solutions. Authors in [12] proposed a new heuristic algorithm which can provide full coverage as it also considers the capacity dimension. This capacity dimension is based upon the different spreading factors and also the channel utilization. The proposed algorithm was evaluated in real environment and in different random topologies to observe its outcomes. In addition to that, their investigation showed that it can achieve full coverage with low number of gateways while considering the maximum achievable number of nodes per access point.

Authors in [13] also proposed a new gateway placement model to effectively place the multiple gateways into the LoRaWan network. This model, in contrast to [12], separates the IoT devices into groups based on similarities aiming to offer the best possible service. They evaluated their work in a simulation where the results proved its benefits in contrast to other placement models.

Further, in [14] authors proposed an efficient planning placement algorithm for LoRaWAN networks. A mechanism is also presented for an optimal nodes’ configuration. They evaluated and confirmed their efficient solution in a simulator using different networks.

## 3. FUOTA Using Multiple Gateways

### 3.1. Firmware Update Process

The firmware update process is a composite procedure that involves a series of steps before completion. The first step is to identify devices that need a firmware update and generate the multicast groups. Each multicast group is associated with one or more gateways and consists of several devices that can receive data simultaneously. The use of radio multicast communications greatly improves the network’s performance. Instead of sending the same frame to each device individually, each frame is sent only once and can be received by all devices in the community at the same time. However, to accomplish concurrent data acquisition, a couple of steps must be performed before proceeding. As a reminder, the LoRaWAN protocol supports three classes A, B, and C, each one offering a different trade-off between latency and battery life.

Class A is considered power-efficient with high latency since two downlink intervals are followed only after an uplink transmission. Class B is less power-efficient but can attain less latency than class A as more downlink programmable intervals are scheduled. Finally, class C, even if it is power inefficient, can accomplish extremely low latency since a device continuously listens for downlink info as it has incessantly open downlink slots. A device stops to listen during uplink transmission. Consequently, devices must switch from class A to class B or C temporarily to receive the firmware image. Then, they must be synchronized according to a clock to receive the data concurrently. The generation of the firmware image is then performed and signed to preserve the authentication and integrity. Further, this firmware image is transmitted to the network where devices in multicast groups receive the image and verify and install the new firmware.

**Figure 1:**
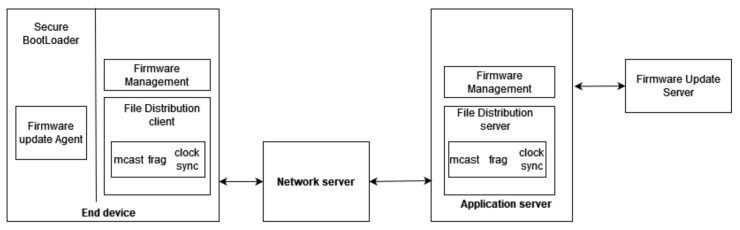
FUOTA architecture.

Figure 1 describes the FUOTA architecture where interfaces with solid lines are described and handled by the LoRa alliance specifications. Otherwise, they are out of scope of Lora Alliance.

On the right side of Figure 1, the Firmware Update Server interfaces with the Firmware management module and generates the firmware image for a list of devices and selects fragmentation parameters such as the number of fragments, redundant fragments, etc. This image is then signed using the FUS private key to maintain confidentiality and integrity. The file distribution server uses the new features multicast, fragmentation, clock synchronization, etc., to attain the firmware image’s delivery to the multicast group.

LoRaWAN uses the duty cycle to keep the channels’ fairness. By transmitting a specific code individually to several field devices, the network system would be suspended for a long time. Hence, the LoRa alliance proposed the multicast mechanism where devices can receive the code simultaneously, leading to an optimized use of the duty cycle and a reduced network interruption time. To achieve multicasting, devices must be synchronized to receive the code simultaneously, and thus a clock-synchronization mechanism was included. As the code consists of few hundreds of kilobytes and LoRaWAN only supports low payload sizes, fragmentation was essential to break the code into equal fragments.

Note here that multicast, clock-synchronization, and fragmentation were not enough to implement the FUOTA procedure. To this end, new additional mechanisms for sending a downlink transmission without the need for uplink transmission and recovery from packet losses without re-sending the packets were developed in order to ensure an efficient FUOTA procedure.

To continue with, the Network server located in the middle of Figure 1 is responsible for several things concerning the FUOTA procedure. It can generate, delete, or modify the multicast groups and relay packets from LoRaWan devices to the application server and vice versa. In addition to that, it delivers the firmware image to the multicast groups.

Once an end device receives enough fragments from the NS, it reconstructs the binary image, and then it performs some security tests. These tests include the sender’s authentication using the FUS’s public key, which is already stored in all devices. The counterparts on the devices’ side are presented on the left side in Figure 1, the file distribution client, and the firmware update agent, along with a secure bootloader. The bootloader is the most crucial part on the device’s side since it completes the FUOTA process. It checks the availability and integrity of firmware upgrade images, decompresses them, and overwrites any previous firmware image.

### 3.2. Firmware Update Using Multiple Gateways

The FUOTA process must be completed quickly to reduce the systems’ interruption and at the same time to update the maximum number of devices. In this way, the highest system performance is achieved.

To carry out this complicated task, the lowest allowed spreading factor (SF = 7) should be used. Although lower spreading factors provide higher data rates and payload sizes, leading to faster procedures, they attain small distances. Thus, if the distance between the GW and the device is greater than the propagation range, the device will not be able to receive or transmit any network information. Therefore, a single GW can lead to lower update efficiency considering the FUOTA procedure if a high subsection of devices are located in greater distance than the transmission range. On the other hand, higher spreading factors can achieve greater update efficiency since longer distances can be accomplished. As a reminder, high spreading factors are more resilient to noises and interference and therefore can attain extensive ranges. Nevertheless, the former results in high energy consumption since the activation time is increased. In addition to that, the update time is also raised substantially since higher spreading factors result in higher time on air. To have a better understanding on the impact that spreading factors have on firmware update process, consider transmitting 100 kbytes firmware image using two different data rates, DR0 (S12, BW125 kHz) and DR5 (S7, BW125 kHz). In order to successfully update the devices it would require 2008 packets when using DR0 and only 461 downlink packets when using DR5. In addition to that, since the air time for DR0 (2793.5 ms) is much higher than in DR5 (368.9 ms), it would require a significant amount of time to transfer packets using DR0 compared to DR5. Nevertheless, since many systems require small outages, this high intervention is considered an unacceptable case for many applications. The trade-offs mentioned above were confirmed in [4,11] when the FUOTA procedure was performed using a single GW.

Multiple gateways are used in this work to overcome these limitations and offer the highest efficiency, along with the highest data rate (DR5). As it is proven from the literature, the packet delivery ratio (PDR) is affected dramatically by the communication range. Multiple gateways can increase reliability, improve the coverage, and overhaul the trade-offs mentioned above since they reduce the transmission range substantially as devices are more likely to be within a gateways’ distance. As the communication distance is smaller, the packet error rate is reduced dramatically due to the high possibility of receiving the frames. Hence, this leads to an improved packet delivery ratio (PDR). Further, higher data rates can be applied to reach the destination nodes. In consequence, this lower distance, along with higher data rates, can significantly increase the update efficiency and, at the same time, reduce the update time and power consumption.

### 3.3. Multiple Gateways Simulation Scenario

In this work, we extend the functionalities of the FUOTAsim simulation tool [4] to support a firmware update scenario with multiple gateways. The main addition to the FUOTAsim simulator was the integration of the Okumura-Hata model to simulate a city’s propagation model. The supported features of this tool, such as the duty cycle restrictions and the Okumura-Hata model, lead to more realistic simulation results [4]. The principal purpose was to identify the maximum communication distance to maintain the communication link between the node and the gateway, and therefore to use this distance to create a number of clusters in the simulator. Using clusters, we can simulate the FUOTA requirement of multicast groups during the firmware update procedure.

Our scenario simulates a city environment with 10,000 nodes distributed randomly, following a uniform distribution, in a square area of 10 km × 10 km.

#### 3.3.1. Propagation Model

For this paper, a different path loss model was selected called Okumura-Hata, in contrast to [4]. Okumura-Hata is a well-known radio propagation model and there are many papers about LoRaWAN which use it to obtain the numerical results such as [15,16,17,18,19].

This model can be utilized in typical urban areas, for instance, large or small-medium-sized cities, in suburban or rural areas. It is widely deployed as it estimates the effect of the propagation mechanisms: scattering, reflection, and refraction. It also considers other metrics that influence the propagation, such as the antenna height of nodes (end nodes and GW) and distance between the end nodes and the GW. Therefore, it contributes to more realistic simulation results.

In general, the path loss in Okumura-Hata model is written as Equation (1):

(1) PL=A+B×log(d)+C

where *A*, *B*, and *C* are factors that depend on frequency and antenna height:

(2) $$A=69.55+26.16\times log\left(f_{c}\right)-13.82\times log\left(h_{b}\right)-a\left(h_{m}\right)$$

(3) $$B=44.9-6.5\times log(h_{b})$$

where $f_{c}$ is the carrier frequency(MHz), *d* distance between mobile node and base station, $h_{b}$ base station height, $h_{m}$ node antenna high.

In our scenario, we use the urban model for small-medium-sized cities where the Okumura-Hata model is formulated as:

(4) $$a\left(h_{m}\right)=\left(1.1\times log10\left(f_{c}\right)-0.7\right)\times h_{m}-\left(1.56\times log10\left(f_{c}\right)-0.8\right)$$

In the case of small and medium-size cities, C=0.

Using Equations (1)–(4) where $h_{b}=20$ m, $h_{m}=3$ m, $f_{c}=868$ MHz, and maximum path loss to maintain connectivity to be $PL_{max}=142$ dBm, we calculate the maximum transmission range for our setting. The value of $PL_{max}$ is based on LoRaWAN data rate and is given by Equation (5).

(5) $$PL_{max}=TransPower+Gains-Losses-Rx.Sensitivity$$

where TransPower=14 dBm, Gains=5 dB, Losses=2 dB and Rx.Sensitivity=−125 dBm (for SF7). The receiver sensitivity is given by Equation (6).

(6) $$Rx.Sensitivity=-174+10log10\left(BW\right)+NF-SNR_{Limit}.$$

where BW= 125,000 Hz, NF=6 dB and $SNR_{Limit}=-7.5$ dB for SF = 7.

#### 3.3.2. Creation of Clusters

**Figure 2:**
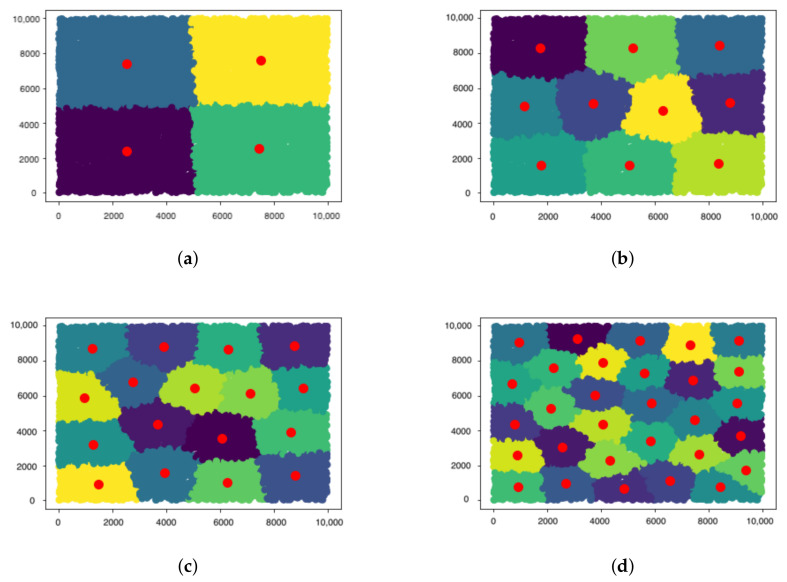
Gateway Clusters (**a**) 4 Gateways, (**b**) 10 Gateways, (**c**) 17 Gateways, and (**d**) 30 Gateways.

Clusters were used to implement the multiple gateways scenario and refine the FUOTA protocol. Clusters can create N groups, based on different factors such as distance, density, etc. For this work, K-means clustering was used, which allows for the generation of k centroids since it works on the principle that a node with the shortest distance from others becomes the centroid, reducing the propagation range. In addition, each node is connected and grouped with the centroid that is closest to it. Each centroid is a GW that is linked to a collection of devices known as multicast groups. The multicast groups and their associated GW are shown in Figure 2.

## 4. Performance Evaluation

To evaluate the impact of multiple gateways over FUOTA process we extended the FUOTAsim simulator [4]. The extension of the simulation tool as described in Section 3.3 leverages the Okumura-Hata path loss model and the K-means clustering algorithm. To produce real LoRaWAN measurements we added a common standard deviation [20] (6.9 dB) into the Okumura-Hata model. Real life plots aim to avoid any unnecessary obstructions in transmission and thus, to fit into real scenarios the gateway was configured in 20 m height. To monitor environmental factors such as air pollution, devices need to be placed in high buildings. Hence, since it is assumed that nodes in the simulator are environmental sensors, they were configured in 3 m height.

In the simulation scenario, it is presumed that the initialization phase was performed and devices are able to retrieve the fragments. Each node in the network uses Class C mode, where their downlink slots are continuously open, allowing a continual data acquisition. Each gateway in the simulation consists of a multicast group where its associate nodes are able to obtain the firmware image. It is assumed that gateways transmit the firmware image simultaneously. The maximum transmission range where a node is located in order to successfully receive fragments is 2051 m, as emerged from the scenarios. Note here that the employed type of antenna for our devices is assumed to be a common omnidirectional antenna with a gain of 2.5 dBi. In addition to this, losses derived from the used cables and connectors of each antenna are assumed to be 1 dB, which is a usual value in real life measurements.

The FUOTA process can take the advantage of redundant fragments to increase the performance. Therefore, different firmware sizes along with different redundant fragments were used to evaluate their impact. Table 1 encapsulates all the simulation parameters that were used for the assessment. Each simulation scenario was performed 10 times using different random seeds and the mean values from each simulation scenario were derived.

The evaluation considers the following metrics in the multiple gateways scenarios:

- Update efficiency indicates the percentage of devices that updated their firmware successfully.
- Update time indicates the firmware update time.
- Lost fragments indicates the number of lost fragments during the firmware transmission.
- Network energy describes the node’s power consumption during the firmware process.
- Corrupted Fragments indicate that a node received a corrupted fragment.

In this investigation we analyze the impact of multiple gateways along with different number of nodes, multiple firmware sizes, and redundant fragments to observe the network’s behavior during the firmware update process.

### 4.1. Evaluation of Multiple Gateways and Firmware Size

In this subsection, we explore the effect of multiple gateways and firmware sizes on the device energy consumption, update efficiency, update time, and the number of corrupted and lost fragments.

**Figure 3:**
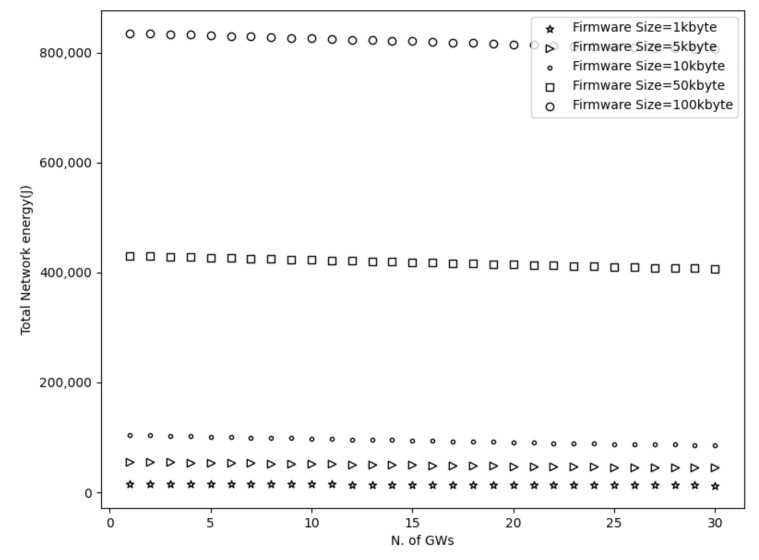
Network energy using multiple firmware size.

**Figure 4:**
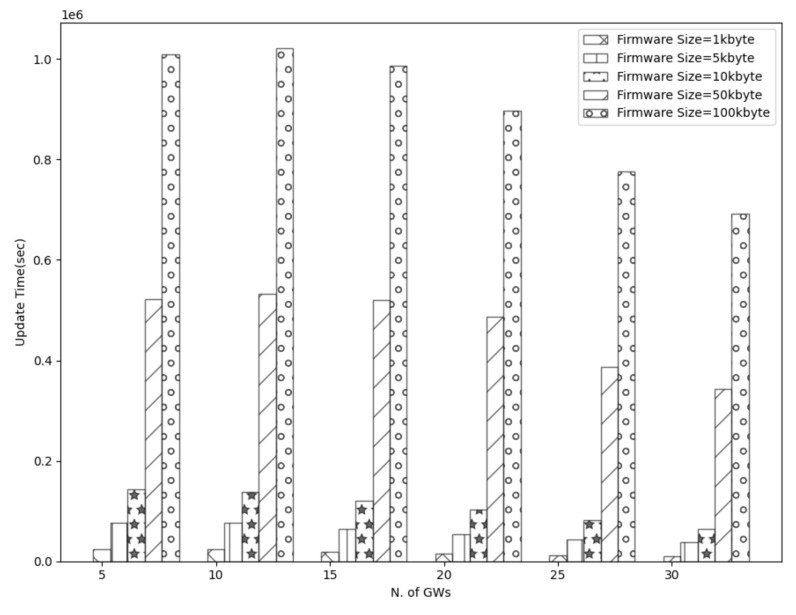
Update time using multiple GWs and firmware size.

**Figure 5:**
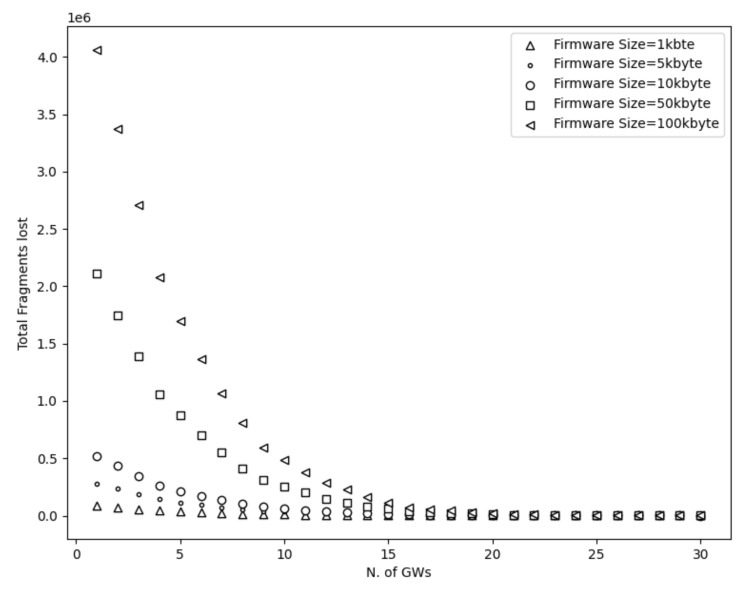
Fragments lost using different firmware sizes.

**Figure 6:**
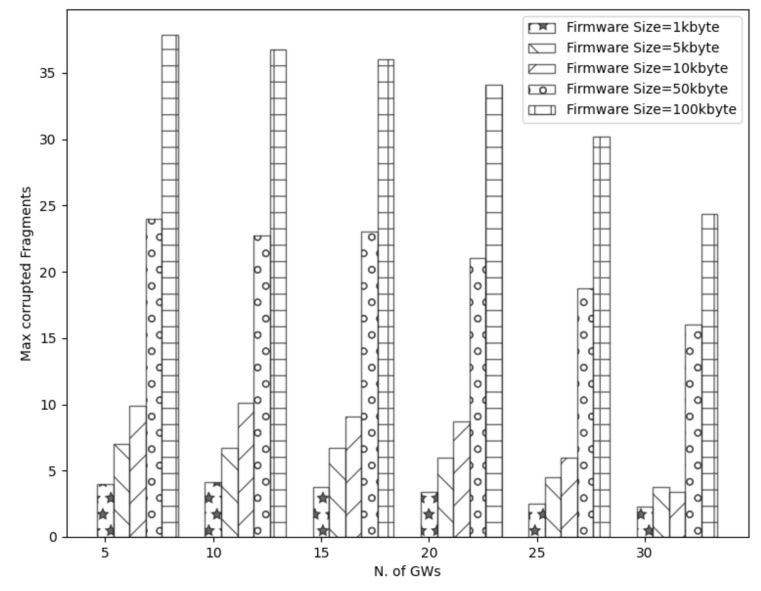
Effect of multiple GWs and firmware size in corrupted fragments.

To start with, Figure 3, Figure 4, Figure 5 and Figure 6 show a decrease in the network energy, update time, and number of loss and corrupted fragments as the number of gateways increases. More gateways can substantially reduce the transmission range, increasing the probability of receiving the transmitted fragments correctly. Consequently, the number of lost and corrupted fragments is reduced dramatically. Therefore, as the network experiences fewer losses and corruptions, lower device energy consumption is achieved and shorter total update time is attained.

**Figure 7:**
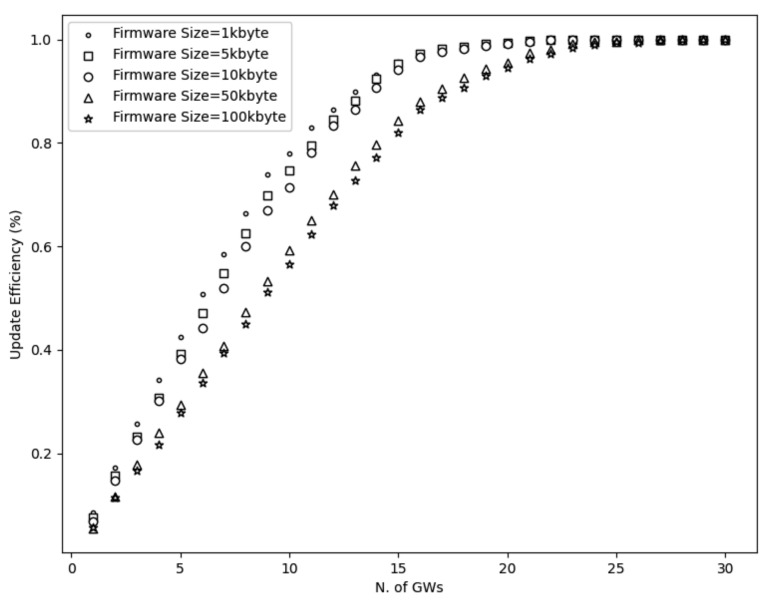
Update efficiency using 10,000 nodes and different firmware size.

Further, from Figure 7, it is perceived that with a high number of gateways, a 100% update efficiency can be attained. This is a result of the reduced number of lost fragments (almost zero) and the higher SNR as the distance decreases. Therefore, the multicast groups are more likely to receive the firmware image, update their firmware, and accomplish maximum update efficiency. On the other had, and not unexpected, fewer gateways result in higher power consumption, update time, number of lost and corrupted fragments, and lower update efficiency. This is a consequence of the high communication range, which strongly impacts the firmware update process. As a reminder, the lowest spreading factor (SF = 7) is used, which performs better with small distances since it has lower noise immunity than higher spreading factors. Hence, the transmitted signal would experience more attenuation as it travels in excessive ranges. Consequently, devices located far from the gateway may not receive or decode the transmitted fragments successfully. Therefore, a high number of fragment corruptions or fragment losses would be obtained, and thus, lower update efficiency, high energy usage, and more update time would be realized. The aforementioned results are confirmed in Figure 3, Figure 4 and Figure 7.

Considering the firmware size as shown in Figure 3, Figure 4 and Figure 5 and Figure 7 and Table 2, higher firmware size can also affect negatively the network. When a larger firmware size is used, multicast groups need to retrieve many fragments to complete the firmware update. Hence, as the firmware size is increased, the power consumption, number of lost and corrupted fragments, and update time rise as well. As a means of example, a firmware update process using 1 kbyte and 5 GWs requires more than a day to complete, in contrast to 100 kbyte firmware size, which requires more than 11 days. Nevertheless, as the number of GW increases, the update time for all the firmware sizes decreases. For the maximum number of simulated GWs (e.g., 30), the update time is reduced to 2 h and 19 h for 1 kbyte and 100 kbyte, respectively. In addition, to obtain the greatest update efficiency, more gateways are needed (≥23 see Figure 7) in contrast to smaller firmware sizes that can achieve 100% update efficiency using a lower number of gateways (≥20). However, it is perceived that in a great number of gateways, the update efficiency and also the number of lost fragments (Figure 5) stabilizes and attains maximum performance. As the distance is reduced, the signal strength becomes stronger, having as a result to be successfully decoded as it can easily reach the destination nodes. Subsequently, it is acquired that the large firmware sizes do not affect the update efficiency and the number of lost fragments in a high number of gateways, but it only affects the power consumption and the total update time. Combining high firmware size with a small number of gateways also has a dismissive impact, as results showed. The excessive communication distance along with higher number of fragments in order to complete the firmware update can cause the highest energy consumption, update time, lowest update efficiency, and highest number of corrupted and lost fragments as shown in Figure 3, Figure 4 and Figure 5 and Figure 7 and Table 2 respectively.

Therefore, it is derived that the firmware size, transmission range, and the number of gateways have a crucial impact on the network results.

### 4.2. Evaluation of Multiple Gateways and Network Size

**Figure 8:**
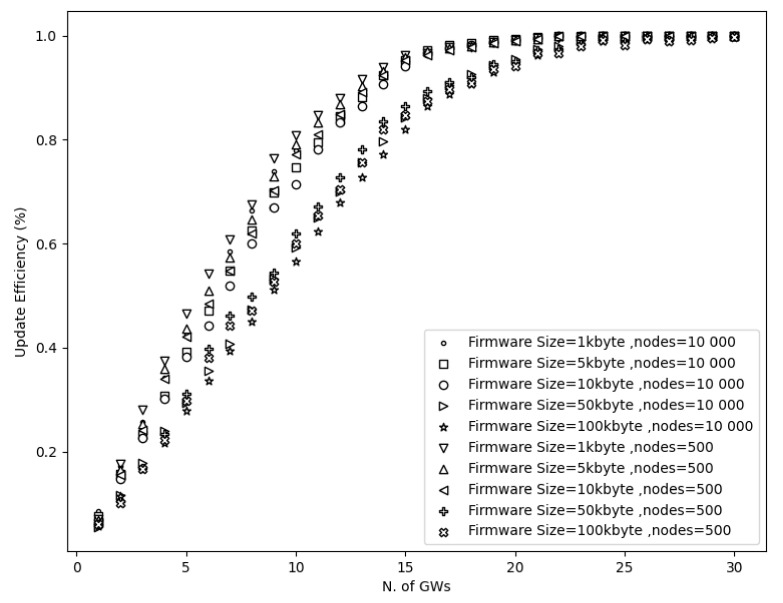
Update efficiency vs. multiple GWs and number of nodes.

Besides the firmware size, it was important to investigate the impact of multiple gateways and a different number of nodes during the firmware update process. Results in Figure 8 show that the number of nodes does not affect the update efficiency. As a reminder, the update efficiency shows the percentage of the updated devices. This percentage depends only on the transmission range and firmware size, which can negatively affect the update efficiency as these parameters escalate.

**Figure 9:**
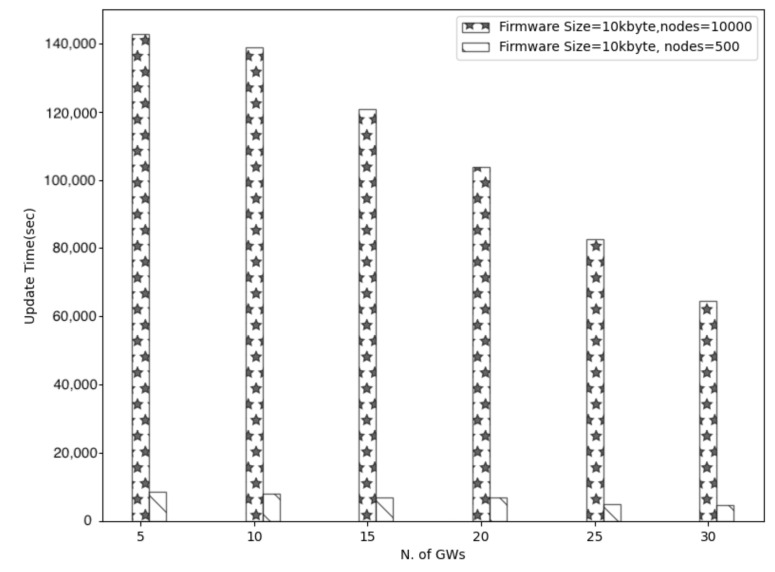
Update time vs. multiple Gws and number of nodes.

On the other hand, the network size influences the total update time remarkably. The total update time represents the required time to complete the update process. To put it simply, it constitutes the maximum duration that devices need to accomplish the update. Figure 9 shows that the update time is proportional to the network size and grows about 20 times when 10,000 nodes need an update instead of 500. However, as Figure 9 shows, multiple gateways can reduce the total update time for all the network sizes.

### 4.3. Evaluation of Multiple Gateways and Redundant Fragments

As a reminder, a gateway transmits the firmware image to a multicast group, and devices can receive the fragments simultaneously. However, due to weak signal strength emerging from excessive distances or possible interference coming from different environmental factors, only a subsection of the multicast group receives each fragment correctly. Hence, these lost or corrupted fragments would need to be re-transmitted. Subsequently, many re-transmissions are required in order for all the devices to receive the fragments correctly, and this number increases as the number of devices rises [21]. To overcome this high re-transmission problem, a new method was considered, called redundant fragments. Redundant fragments can reconstruct any lost or corrupted fragment and fulfill the firmware update process.

More specifically, the Lora alliance defined a simple Forward Error Correction (FEC) code that must be applied before sending the fragments [22]. In that way, devices can autonomously reconstruct any lost or corrupted fragment and fulfill the procedure without requesting any re-transmissions. In addition, an arbitrary redundancy can be selected from the transmitter. For example, by selecting 20% redundancy, the receiver is allowed to lose roughly 20% of the frames and still be able to reconstruct the firmware image.

The firmware image must break into equal fragment sizes to support the payload limitations. These chunked fragments are labeled as uncoded fragments [22]. To allow the original reconstruction of the firmware image, the sender must transmit more coded fragments than the total number of uncoded messages. Therefore, for N uncoded fragments, there is a need to send N+M coded fragments [21]. The coded fragments are derived by performing an XOR operation with some of the uncoded fragments. In that way, devices can reconstruct any lost packet and finish the update procedure.

**Figure 10:**
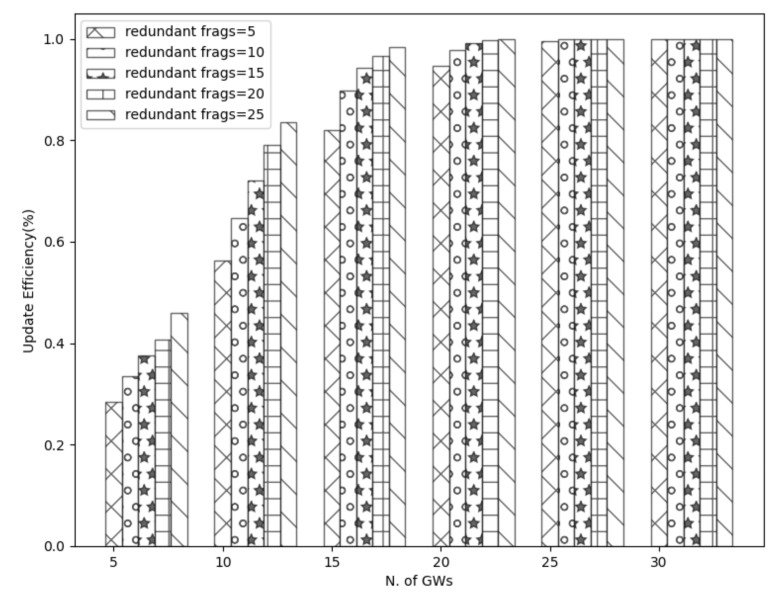
Effect of redundant fragments in update efficiency.

Figure 10 shows the trend mentioned above where the update efficiency is higher when applying more redundant fragments. For example, using 25 redundant fragments instead of 5 along with only 5 GWs, the achievable update efficiency is increased by approximately 20%. Combining multiple gateways with redundancy, the performance is ameliorated dramatically. By utilizing only 15 gateways along with a high number of redundant fragments, the update efficiency is almost (100%). As the distance is reduced, the packet error rate decreases, and thus, there are fewer lost or corrupted fragments. Further, nodes can reconstruct any corrupted or lost fragment because of the redundant fragments. To continue with, it is perceived that the number of redundant fragments does not affect the update efficiency when the number of gateways is high; as explained before, this is because the signal is stronger, and therefore devices receive the fragments correctly. Hence, the redundant fragments are essential only in a small number of gateways and when the distance is high.

**Figure 11:**
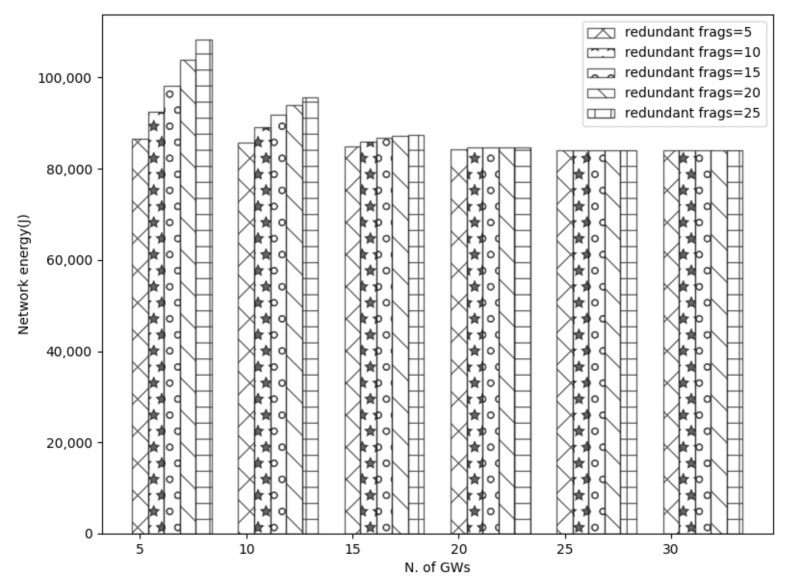
Effect of redundant fragments in network energy.

However, a higher number of redundant fragments result in higher network energy since more fragments are processed. This is shown in Figure 11 where a higher redundancy causes more battery drain. As a means of example, using 25 redundant fragments instead of 5 with 5 GWs, the network energy increases by about 30%. However, as the number of gateways increases, the network energy decreases for all the redundant sizes. Further, it is observed that the network energy stabilizes when using ≥20 gateways. Again this effect can be explained from the reduced distance where a lower communication range results in a higher possibility of receiving the fragments. Therefore, the redundant fragments are not utilized.

Considering the above results, the update efficiency needs to be balanced with the battery consumption and deployment cost.

## 5. Discussion

In this section, we present some further conclusions from the simulation results. Next, we discuss some ideas on how to reduce the power consumption. The importance of greater network planning, the reduction of possible downlink/uplink collisions, and a realistic setup considering the current framework’s complexity along with possible limitations or optimizations are discussed as well.

### 5.1. Extend Device Lifetime

Simulation results showed that multiple gateways could reduce network energy even if higher firmware or redundant fragments are used. Large firmware sizes and many redundant fragments can negatively affect the system since a higher number of fragments must be processed. According to the simulation results, as the number of gateways increased, the network energy decreased, resulting in a greater device’s lifetime. More gateways can reduce the transmission range substantially, and therefore this increases the probability of receiving the transmitted fragments correctly. Hence, the network experiences fewer losses or corruption, and thus fewer processes must be performed to attain the update. In addition to that, results revealed that the number of redundant fragments did not affect the network energy when many gateways were used since the energy was stabilized. Nevertheless, it is crucial to mitigate this energy consumption as much as possible to achieve maximum device lifetime. Therefore, class B can be used instead of class C. Class B consumes less energy than class C since the downlink slots are open only in scheduled intervals. Consequently, by combining multiple gateways along with class B, the energy can be reduced the most.

### 5.2. Importance of Gateway Placement

Based on the current simulation scenarios, the derived minimum set of gateways can be used to provide full coverage and preserve acceptable operation and deployment costs while it offers maximum performance. According to the simulations results, 18 GWs can cover a 10 × 10 km area and accomplish a great update efficiency almost 100% for all the firmware sizes. However, simulation results showed that the firmware size affects the update efficiency when a low set of GWs (≤22) is used. More specifically, with ≤22 GWs one can accomplish a higher update efficiency when lower (1, 5, and 10 kbyte) firmware size is used in contrast to higher firmware sizes (50 and 100 kbytes) as shown in Figure 8. Nevertheless, as the GW diversity increases (≥23), a 100% update efficiency can be accomplished since there are not any lost fragments (see Figure 5). As a reminder, this is because the communication distance is reduced substantially and hence this results in a higher possibility of receiving the fragments. Therefore, it is derived that the firmware size does not affect the update efficiency when a high number of GWs are used.

Despite the above mentioned, it is essential to consider other parameters such as the manufacturing cost, possible limitations due to the duty cycle, and the maximum number of devices per cell to diminish the collisions as much as possible. Therefore, it is crucial to improve the current gateway placement.

### 5.3. Reduction of Downlink/Uplink Collisions and Interference

The reduction in the transmission range leads to coexistence considerations and issues that can occur in the future. As the communication distance is reduced and gateways are closer, interference can occur when the same or adjacent frequency is used. Hence, concerning the FUOTA process, an interpose can lead to higher power consumption and update time since more re-transmissions would be required. Thus, it is essential to reveal and mitigate those collisions as possible to preserve the most efficient system performance that multiple gateways confirmed to offer during the firmware update process. In addition, authors in [5] confirmed that uplink collisions would occur as well since several devices would use the same parameters (SF7, BW125 kHz) as the transmission range is reduced. LoRaWAN exhibits the capture effect where only the strongest signal survives and hence, if the same parameters are used, the weaker signal would be suppressed by the strongest. Nevertheless, uplink collisions can be reduced by using an efficient adaptive data rate algorithm that can offer high reliability and throughput.

### 5.4. A Realistic Setup

Besides the results from the simulator, it is also essential to consider the complexity of the above framework and possible limitations or optimizations in a real operational environment before the implementation.

Our experiments demonstrate the minimum set of GWs that can attain best performance using different firmware sizes. However, this great network performance comes at the expense of the deployment cost. As previously mentioned, simulation results revealed that to accomplish maximum update efficiency, full coverage of a small-medium sized city of 10 × 10 km and preserve acceptable operation and deployment costs, 18 GWs are needed. Nevertheless, in exchange for update efficiency, a lower number of GWs can be used to mitigate as possible the manufacturing cost. As a means of example, 15 GWs update more than 80% of the total number of devices. A way to reduce the manufacturing cost and increase at the same time the update efficiency is to use redundant fragments to fulfill the FUOTA procedure. However, as it was derived, the number of redundant fragments affected negatively the network energy when a small-scale network(<15 GWs) was utilized. In higher number of GWs redundant fragments were not utilized since the communication range was decreased which resulted in higher possibility of receiving the fragments. Furthermore, the manufacturing cost can be even more reduced if a higher spreading factor is applied along with an increased transmission power due to the greater communication link. According to simulation results, the transmission link using DR0 is 4384 m compared to DR5, which is 2051 m.

Nevertheless, this comes at the expense of a raised update time and devices’ energy consumption. However, as this work showed, these parameters must be optimized to accomplish best performance when using the FUOTA procedure. A practical solution is to improve the current GW placement, apply the Class B, and use an efficient ADR algorithm as described in Section 5.1, Section 5.2 and Section 5.3 in order to update the maximum number of nodes per cell using the highest possible data rate and decrease possible uplink or downlink collisions while mitigating the utmost operational and deployment cost. Nonetheless, the above mentioned solution requires a further analysis that is out of this paper’s scope and is considered future work.

Another feature that must be considered is the need for uplink transmission during a firmware update. As a reminder, A LoRAWAN node employs a bi-directional communication, which can perform an uplink or a downlink transmission in a fraction of time. Further, a GW is not able to receive any data while transmitting. Considering the duty cycle restrictions, GWs are not able to acquire data for 10% of the time. However, this high interruption is crucial for many applications. A solution is that GWs will acquire and transmit data in a round-robin fashion. As literature shows [23], uplink and downlink collisions will not collide even if nodes use the same parameters such as SF, frequency, and bandwidth. The GW uses I/Q signals while transmitting, which ensures that its transmission will not interfere with that of an end node [23].

## 6. Conclusions

In this work, we performed a comprehensive evaluation of the performance of the firmware update over the air during a multiple gateways scenario. Results showed that gateways have a significant role in the FUOTA process since they reduce the transmission range, thus impacting the system’s performance. As the distance is reduced, a higher number of gateways can reduce the network energy, accomplish 100% update efficiency, and attain a short update time since the loss or corrupted fragments are mitigated. We also investigated the effect of multiple gateways and different network and firmware sizes to evaluate their impact on the system performance during the FUOTA process. Simulation results revealed that the former parameters affect the overall performance. We plan to optimize the placement of the gateways for future work and use class B to further reduce power consumption and define possible collisions in the network. 

## Figures and Tables

**Table 1:** Scenario parameters.

Parameter	Value
Number of Gateways	1–30
Area (km)	10 × 10
Number of Nodes	500 and 10,000
Firmware Size (kbytes)	1–100
Okomura-Hata Settings	Gateway Height = 20 m Node Antenna = 3 m
Maximum Transmission Range (m)	2051
Spreading Factor	SF7
Fragment size(bytes)	222
Redundant Graf	5
Random seeds	1000
Class	C
Frequency (MHz)	869.525
GWs Antenna Gains (dB)	5
GWs Transmission Power (dBm)	14
Antenna Losses (dB)	2

**Table 2:** Max corrupted fragments using multiple firmware sizes.

Firmware Size (kbytes)	Max Corrupted Fragments
1	4.2
5	7.4
10	10.3
50	24.5
100	39.11

## Data Availability

Not applicable.
